# Improving Bonding Durability in Dental Restorations: The Impact of Bioactive and Reinforcement Particles on Universal Adhesives

**DOI:** 10.3390/ma18194433

**Published:** 2025-09-23

**Authors:** William Cunha Brandt, Isaías Donizeti Silva, Andreia Carneiro Matos, Flávia Gonçalves, Leticia Boaro

**Affiliations:** 1 Faculdade de Odontologia, Universidade Santo Amaro, Av. Prof. Eneas de Siqueira Neto, 340, São Paulo 04829-900, Brazil; williamcbrandt@yahoo.com.br (W.C.B.); isaiasdonizeti@gmail.com (I.D.S.); andreiacarneiro@gmail.com (A.C.M.); flgoncalves@prof.unisa.br (F.G.); 2 College of Dentistry, University of Saskatchewan, 107 Wiggins Rd, Saskatoon, SK S7N 5E5, Canada

**Keywords:** dental adhesive, bioactive particles, chlorhexidine, silica nanoparticles, bond strength, antimicrobial, degree of conversion, universal adhesive

## Abstract

Objective: This study aimed to evaluate the effect of incorporating bioactive particles (montmorillonite loaded with chlorhexidine, MMT/CHX) and different concentrations of silica nanoparticles (0%, 3%, 5%, 7%, 10%, and 15 wt%) into a universal dental adhesive on its degree of conversion, bond strength, water sorption, solubility, and antimicrobial activity. Materials and Methods: A universal adhesive was modified with 1 wt% MMT/CHX and varying amounts of silica nanoparticles. Degree of conversion was analyzed by Fourier transform infrared spectroscopy (FTIR), and microtensile bond strength was evaluated at 24 h, 6 months, and 12 months after water storage. Water sorption and solubility were measured according to ISO 4049, and antibacterial activity was tested against *Streptococcus mutans* using the agar diffusion method. Results: All experimental adhesives containing ≥7% silica showed significantly reduced water sorption and solubility. The presence of MMT/CHX imparted consistent antimicrobial activity across all experimental groups. Degree of conversion remained stable across all groups and storage periods. Notably, after 12 months, only the experimental groups maintained or improved bond strength, while the control group showed a significant reduction. Failure mode analysis indicated increased mechanical integrity with higher filler content. Conclusions: Incorporating 1 wt% MMT/CHX and ≥7 wt% silica into a universal adhesive improved long-term bond strength, reduced degradation, and introduced antibacterial properties without compromising polymer conversion. These findings support the potential of developing durable, bioactive adhesive systems for restorative dentistry. Clinical Significance: The incorporation of bioactive and reinforcing nanoparticles into universal adhesives enhances bond durability and introduces antibacterial properties without compromising polymerization. This innovation may lead to longer-lasting restorations and reduced risk of secondary caries in clinical practice.

## 1. Introduction

The advancement of adhesive dentistry has facilitated the development of more conservative restorative techniques, as well as the establishment of effective bonding between tooth structure and restoration [1,2,3]. To achieve this, three-step adhesive systems that utilize prior acid conditioning of dentin and enamel are considered the gold standard for ensuring adequate bonding of resin-based restorative materials to the dental substrate. According to various studies, this system provides high values of bond strength [4,5]. However, over time, the technique of acid conditioning of dentin can lead to increased permeability of the hybridized dentin, greater degradation of the bond, and, consequently, a reduction in bond strength [6]. This is primarily due to the activation of matrix metalloproteinases, which degrade dental collagen, thus impairing the tooth/restoration bond [7].

In order to reduce the activation of metalloproteinases that degrade the bond and also decrease clinical time for the fabrication of adhesive restorations, adhesive systems have been simplified. “Universal” single-bottle adhesives have been developed. In this adhesive system, the acid primer and adhesive resin are combined in a single bottle, reducing the adhesive application technique to one step [8,9].

Nonetheless, dental adhesives remain the weakest link in dental restorations, contributing to marginal gaps and consequently the development of secondary caries [10,11]. This limitation in bonding systems can lead to the need for restoration replacements and, therefore, the loss of healthy dental tissue. This problem may arise due to the low mechanical strength of adhesives when subjected to stress generated by polymerization shrinkage of composites, as well as the fragility of their polymeric structure, often porous due to the presence of hydrophilic components in their composition [12,13]. As such, in order to improve the durability of the adhesive interface and the mechanical properties of the adhesive interface, inorganic filler particles, ranging in size from 5 to 400 nm, were incorporated into the composition of these resinous materials [13,14,15,16]. These inorganic particles are added with the aim of reducing polymerization shrinkage, increasing cohesive strength, and enhancing the quality of the polymer formed, thereby promoting better sealing of the bonding interface [13,14].

The inorganic filler particles included in small proportions in dental adhesives need to penetrate the dentinal tubules to ensure uniform distribution of the filler particles throughout the adhesive layer and hybrid layer [15,16]. Therefore, it is necessary to incorporate nanometric-sized filler particles into these adhesive systems, with the subsequent possibility of integrating the inorganic particles throughout the entire extent of the applied adhesive resin. A previous study [14] showed that incorporating 10% by weight of 7 nm filler particles into hydrophobic resins of conventional three-step adhesive systems could improve the cohesive strength of this material without affecting the degree of conversion of the resinous polymer structure.

Another factor that may affect the tooth/restoration bond is the degradation of the adhesive bond. Both the adhesive polymer and the collagen present in the hybridized layer are susceptible to degradation. However, collagen degradation remains the primary concern [6]. Studies have shown that the prior application of chlorhexidine can neutralize the matrix metalloproteinases that degrade collagen. Therefore, the development of bioactive adhesives capable of neutralizing metalloproteinases would be of significant importance in increasing the longevity of dental restorations. Consequently, carrier particles such as nano-montmorillonite (MMT), which can release substances that inhibit metalloproteinases, such as chlorhexidine (CHX), may aid in this task [17,18]. This bioactive particle of MMT loaded with CHX has been previously described in the literature and showed promising results, especially in antibacterial activity in different polymeric matrices such as di-methacrylate [17], poly methyl-methacrylate [18], and epoxy resin [19], but is still not being tested in dental adhesive systems.

New formulations of simplified dental adhesives (single-bottle systems), containing varying amounts of nanometric filler particles, are important for evaluating the development of bonding agents with improved physicomechanical properties [20]. Thus, it becomes essential to assess the effect of incorporating different quantities of nanometric inorganic particles and bioactive particles into universal adhesives on the degree of conversion and bond strength, and to analyze the feasibility of developing bioactive adhesives.

Building on the concepts outlined above, the development of a bioactive adhesive system capable of controlled chlorhexidine (CHX) release, reinforced with an increased concentration of filler, holds significant promise for creating durable, long-lasting restorations. This approach not only enhances the adhesive mechanical properties but also offers antibacterial benefits, potentially improving the longevity and performance of dental restorations in the oral environment.

Therefore, the purpose of this study was to analyze (1) the addition of a bioactive particle (MMT/CHX) and (2) the different concentrations of silica nanoparticle (0%, 3%, 5%, 7%, 10%, and 15 wt%) on the degree of conversion and bond strength of a universal adhesive with bioactive properties after 24 h, 6 months, and 12 months of storage.

The null hypothesis is that the incorporation of both particles (silica and MMT/CHX) will not affect the evaluated properties independently of the storage period.

## 2. Materials and Methods

**Figure 1:**
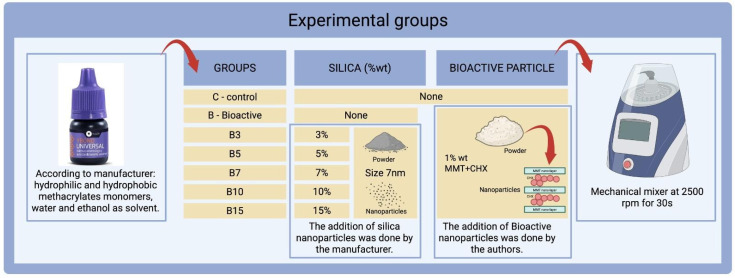
Experimental groups and filler addition. Created in BioRender. Boaro, L. (2025) https://BioRender.com/i62y660 (accessed on 7 September 2025) (Agreement number: ET28QNL37C).

The commercially available universal self-etching adhesive YBond Universal (Yller Biomaterials, Neodent, Pelotas, Brazil) base was used as the standard matrix. According to the manufacturer, its polymeric matrix is composed by hydrophilic methacrylates monomers, hydrophobic methacrylates monomers, initiators, stabilizers, and silane, and it has water and ethanol as solvent. The experimental groups are described in Figure 1. The control group (C) did not receive any particles. The bioactive groups (B-B15) received 1% in weight of MMT/CHX particles. The MMT/CHX particles were synthesized as described before [17,18,19], then briefly CHX was first incorporated into the MMT nanoparticles by dispersing them in an aqueous solution of CHX at a 10% weight-to-weight ratio (*w*/*w*). The mixture was continuously stirred for 3 h at a temperature of 80 °C. Following this emulsion process, the compound was freeze-dried for approximately 6 h. Following this process, the resulting MMT/CHX particles contain 7 wt% of CHX intercalated within their lamellar structure. Different amounts of nanosized silica particles (7 nm, Aerosil 320, Degussa, Germany) were added to the bioactive adhesive by the manufacturer and 1 wt% MMT/CHX particles were added using a mechanical mixer (SpeedMixer DAC 150 FVZ, Hauschild, Farmington Hills, MI, USA) at 2500 rpm for 30 s at 21 °C. Around 10 mL of each adhesive was prepared. No filler agglomeration was visually detected in the samples. All samples were activated using the BluePhase LED light source (Ivoclar Vivadent, Schaan, Liechtenstein).

### 2.1. Sorption and Solubility

Sorption and solubility were determined according to ISO 4049 standards [21]. Cylindrical specimens with a diameter of 15 mm and a height of 1 mm were fabricated using a steel mold (*n* = 5). Photoactivation was performed with a dose of 18 J/cm^2^ (20 s) in each side. A single exposure was able to cover the entire diameter of the samples.

The specimens were then subjected to desiccation in a vacuum desiccator at 37 °C for 14 days. The specimens were weighed on an analytical balance to obtain m_1_. The diameter and height of each specimen were measured to calculate the volume. The specimens were immersed in distilled water at 37 °C for seven days, lightly dried with absorbent paper, and weighed again to obtain m_2_. They were then desiccated as previously described for 14 days and weighed to obtain m_3_. Sorption and solubility were calculated for each specimen using the following formulas:

$$SR=\frac{m2-m1}{V}\;SL=\frac{m1-m3}{V}$$

where *SR* is sorption; *SL* is solubility; m1 is the mass after the initial drying of the specimen (µg); m2 is the mass after the immersion period in water (µg); m3 is the final mass after desiccation (µg); *V* is the specimen volume (mm^3^).

### 2.2. Bacterial Sensitivity Test by Agar Diffusion Technique (Inhibition Halo)

Disc specimens with 5 mm in diameter and 1 mm in height were made using a silicone mold and photoactivation was performed with a dose of 18 J/cm^2^ (20 s). The agar diffusion technique was performed following the recommendations of the CLSI—Clinical and Laboratory Standards Institute (Performance Standards for Antimicrobial Disk Susceptibility Tests: Approved Standard—Eighth Edition, M02-A12) [22]. A bacterial suspension of *Streptococcus mutans* UA159 was adjusted in a spectrophotometer with an absorbance of 0.1 at 660 nm, which is equivalent to 1–2 × 10^8^ CFU/mL. A sterile swab was immersed in this solution and sprayed in a BHI culture plate. Five samples of each material (*n* = 5) were placed on the surface of the seeded plate and incubated in anaerobic conditions at 37 °C for 24 h. The inhibition halo was measured using a digital caliper, in two perpendicular directions. The mean of these measurements was considered the inhibition halo diameter. The samples’ diameters were also measured in order to avoid that small variation in sample diameter affects the result.

### 2.3. Degree of Conversion

For each group (*n* = 10), bar shape specimens (7 mm length × 2 mm width × 1 mm thickness) were made using a silicon mold. The degree of conversion was evaluated by Fourier Transform Infrared Spectroscopy (mid-FTIR, Spectrum 100—PerkinElmer, Shelton, CT, USA), with a coupled attenuated total reflectance element—ATR, which has a horizontal Zinc Selenide crystal (Pike Technologies, Madison, WI, USA) in the center that functions as an active substrate for infrared rays. For the measurement, an unpolymerized drop of the experimental adhesive was initially placed on the zinc selenide crystal of the FTIR device, and then the polymerized specimens were positioned one at a time for the reading. Spectra were obtained before (unpolymerized) 24 h, 6 months, and 12 months after photoactivation with co-addition of 16 scans and a resolution of 4 cm^−1^. The polymerization reaction occurred by the breaking of aliphatic double carbon bonds of dimethacrylates monomers, so the corresponding area under 1638 cm^−1^ was used to calculate the degree of conversion. Also, the absorption band area under the 1608 cm^−1^, corresponding to aromatic double carbon of 2,2-bis [4-[2-hydroxy-3-methacryloxy) propoxi]phenyl]-propane, was used as internal standard in the calculus, since it is not affected by polymerization reactions [23], for uncured and cured materials.

### 2.4. Bond Strength

**Figure 2:**
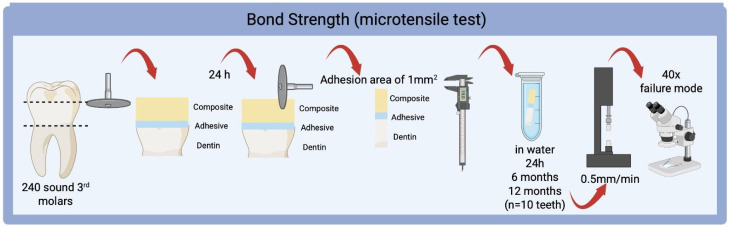
Experimental design for the bond strength. Created in BioRender. Boaro, L. (2025) https://BioRender.com/i62y660 (accessed on 7 September 2025) (Agreement number: ET28QNL37C).

This study was previously submitted and approved by the Research Ethics Committee (CAAE #4,989,582). A total of 210 pristine human molar teeth extracted for therapeutic reasons were selected. Teeth were randomly distributed into the groups (*n* = 10) considering 7 groups of experimental adhesives and 3 different storage periods (24 h, 6 months, and 12 months). Each tooth was sectioned twice according to the method shown in Figure 2, using diamond discs under constant water cooling. The occlusal dentin surface was abraded using silicon carbide sandpaper (#220, 400, and 600) to obtain flat surfaces of medium dentin, and also to simulate a newly formed smear-layer. The experimental adhesives (Figure 1) were actively applied for 20 s, the solvent was evaporated with an air jet, and another layer was applied repeating the same protocol, then photoactivated with a dose of 18 J/cm^2^ (20 s). The restoration was conducted using a composite (Filtek Z350, 3M ESPE, shade A1) with 6 mm in diameter and a thickness of 2 mm using a silicone matrix. Then, a polyester strip was positioned on top of the restoration with the silicon matrix and a 500 g weight was placed on the strip. After 10 s, the weight was removed, and the composite was photoactivated with a dose of 18 J/cm^2^ (20 s). The restored teeth were then stored in water and in a dark environment at 37 °C for 24 h. After this period, the teeth were sectioned in bar shaped specimens with 1 × 1 mm, resulting in an adhesive area of approximately 1 mm^2^. The dimensions of each specimen were measured using a digital caliper to individualize the cross-sectioned area and to use in the bond strength calculus. At least 10 specimens of each tooth were individually stored, and tested in 24 h, 6 months, and 12 months (Figure 2). The microtensile test was performed using a universal testing machine (EMIC DL500, São José dos Pinhais, Brazil) at a speed of 0.5 mm/min. The bond strength was calculated by dividing the maximum load (N) required for failure by the area verified for each specimen and expressed in MPa.

### 2.5. Failure Mode

To analyze the failure mode, the samples were analyzed using an optical microscope at 40× magnification and classified as adhesive, cohesive (in dentin or composite resin), or mixed (combination of adhesive and cohesive).

### 2.6. Statistical Analyses

Data from the sorption, solubility, and inhibition halo were analyzed using a one-way ANOVA/Tukey’s test (*p* < 0.05). Data from degree of conversion and bond strength were analyzed using a two-way ANOVA/Tukey’s test (*p* < 0.05) (factors group and storage period).

## 3. Results

The addition of 7% colloidal silica filler or more was able to significantly decrease the sorption and the solubility of the experimental adhesives in relation to the control group (Table 1, Figures S1 and S2).

**Figure 3:**
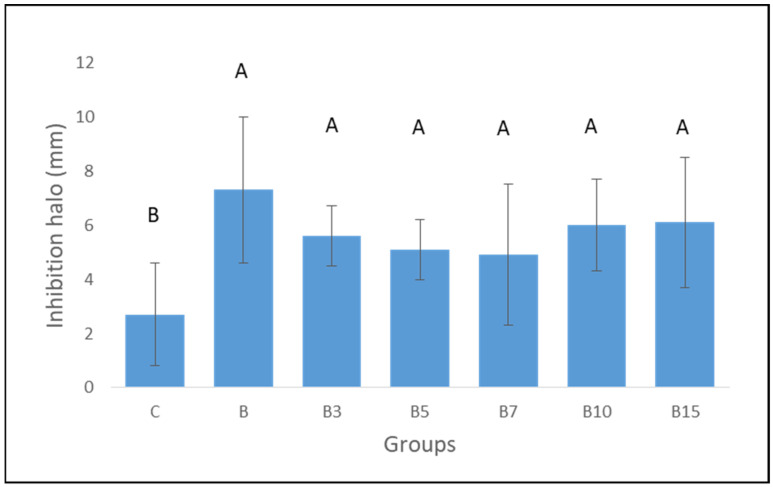
Means and standard deviation of inhibition halo (mm). The letters indicate statistical comparisons among mean. Values followed by the same letter do not present statistical differences (*p* > 0.05).

The presence of 1% MMT/chlorhexidine in all the bioactive materials was able to present the inhibition halo against *S. mutans* independently of the silica concentration. In contrast, the control group, with the absence of MTT/chlorhexidine particles, was not able to generate an inhibition halo against the *S. mutans* (Figure 3).

There is no significant difference in the degree of conversion (DC) of the materials studied at 6 or 12 months (Table 2 and Figure S3). Also, there is no difference between conversion of one material after 24 h and 6 months or 12 months. The only exception is the material with 3% colloidal silica, which presented lower conversion than the material with 7% colloidal silica at 24 h and also presented an increase of conversion from 24 h to 6 months.

The bond strength was similar among all the experimental and control materials after 24 h and 6 months. However, after 12 months the bond strength was higher for all experimental materials in relation to the control. In addition, all the experimental materials were able to maintain the bond strength values along the 12 months except the control materials, which presented a decrease in this property after 12 months (Table 3, Figure S4).

**Figure 4:**
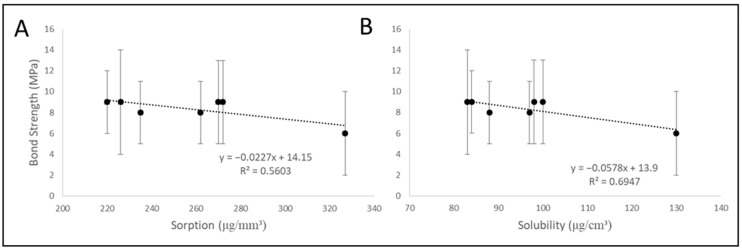
Correlation analysis between (**A**) bond strength at 12 months (MPa) and sorption (µg/mm^3^) of control and bioactive adhesives, and (**B**) bond strength at 12 months (MPa) and solubility (µg/mm^3^).

The correlation analysis among the bond strength at 12 months and sorption and solubility data are presented in Figure 4. There can be observed a moderate correlation of bond strength with sorption (R^2^ = 0.56) and with solubility (R^2^ = 0.69).

When observing the failure mode of the samples in the bond strength test, the cohesive failures are only observed for the control materials and materials with lower filler concentration (B1 to B5) independently of the storage time.

**Figure 5:**
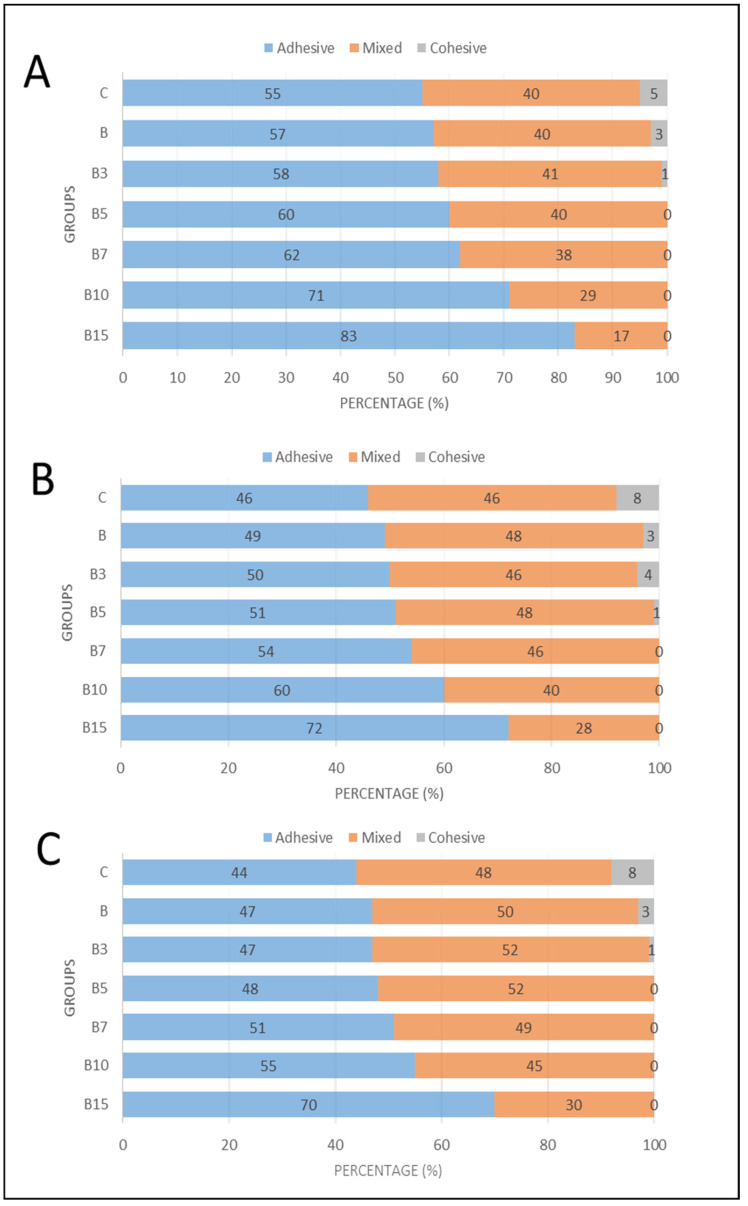
Percentage of adhesive, mixed, or cohesive failure mode after different lengths of time in storage. (**A**) 24 h of storage, (**B**) 6 months of storage, and (**C**) 12 months of storage.

The storage time affected the failure mode (Figure 5). At 24 h the majority of failures were adhesive while at 6 and 12 months the mixed failures were predominant. The difference between the percentage of adhesive and mixed failures is also affected by filler concentration, since the differences are more accentuated in materials with a higher filler amount, independently of the storage time.

## 4. Discussion

In the present study, it was demonstrated that the experimental adhesives suggested were not only able to maintain the bond strength along 12 months, in contrast to the control material, but also presented antimicrobial activity against *S. mutans* without jeopardizing the conversion of the material. Furthermore, the addition of 1 wt% MMT/CHX and 7 wt% colloidal filler or more into the adhesives decreased the water sorption and solubility. So, the null hypothesis that the incorporation of both particles (silica and MMT/CHX) will not affect the evaluated properties independently of the storage period was rejected.

Out of all the adhesives evaluated, only control and experimental were able to maintain the bond strength after 6 months of storage, but only the experimental materials were able to maintain the bond strength after 12 months of storage. In fact, it has been shown that commercial materials present a reduction in bond strength depending on the time [24,25], and it occurs due to two main factors: the degradation of the adhesive layer by the enzymatic action of metalloproteinases [26,27] and by the hydrolytic degradation [27,28]. The experimental adhesives tested acted in both of these factors. It is known that the presence of chlorhexidine is able to inhibit the action of metalloproteinases enzymes [29,30], and apparently the concentration of 1 wt% was satisfactory to this purpose. In addition, the incorporation of filler particles from 7 to 15% significantly decreased the sorption and solubility of the materials, resulting in a decrease in hydrolytic degradation. The correlation analysis among the variables confirms these hypotheses, showing moderated inverse correlations between bond strength and sorption, and also between bond strength and solubility (Figure 3). These results are in agreement with other studies, which observe reduction in hydrolytic degradation in function of the filler amount [27,28].

The failure pattern indicated that increased SiO_2_ filler content enhanced the mechanical properties of the adhesive, leading to fewer mixed and cohesive failures in initial tests (24 h). However, after 6 and 12 months, a general increase in mixed and cohesive failures was noted in all the materials, likely due to degradation in aqueous media, which was able to reduce the adhesive’s mechanical properties over time, even though it did not significantly affect the bond strength. Similar results were observed when PMAA-grafted nanoclay was added in dental adhesives, enhancing microshear bond strength with even small amounts of nanoclay [31].

Another important aspect of the experimental adhesive evaluated is that all presented similar conversion to the control group. Once the degree of conversion is related to cytotoxicity and some mechanical properties [32], the maintenance of the conversion can suggest similar performance. In fact, a related study demonstrated that the inclusion of MMT nanoclays enabled the production of dental composites with low cytotoxicity and reduced polymerization shrinkage [33], without compromising essential physical–mechanical properties like flexural strength, modulus of elasticity, water sorption, water solubility, and hygroscopic behavior. Similarly, it has been reported that resins with MMT/CHX particles exhibited similar conversion to control material without the particles in dental composites and acrylic resin, suggesting that the low filler content had little impact on the polymerization process [34]. In agreement, some authors have demonstrated that particles with size higher than the light wavelength do not interfere in the light scattering and can maintain the material conversion [35].

Finally, it should be highlighted that the presence of 1 wt% MTT/CHX particles guarantees an antimicrobial effect against *S. mutans* in all experimental adhesives, which is an advantage in reducing biofilm growth and can possibly make difficult the development of secondary decay. The antimicrobial effect of MMT/CHX has been observed in previous studies [20,21], either in immediate or late effect [19,36] being observed along 12 months, when added at 5 wt% in dental composites [36].

## 5. Conclusions

According to the limitation of the present in vitro study, regarding the evaluation of only one polymeric matrix and one MMT/CHX concentration, and the absence of mechanical load along the storage, it can be concluded that the addition of 1 wt% MMT/CHX and 7 wt% colloidal silica or more was able to maintain the bond strength of experimental adhesives along 12 months, decrease the water sorption and solubility of materials, and also present antimicrobial activity against *S. mutans.*

## Figures and Tables

**Table 1:** Parameter of sorption and solubility (µg/mm^3^) for control and bioactive dental adhesives (mean and standard deviation). The letters indicate statistical comparisons among mean. In the same column, values followed by the same letter do not present statistical differences (*p* > 0.05).

Group	Sorption (µg/mm^3^)	Solubility (µg/mm^3^)
C—Control	327 (34) a	130 (17) a
B—Bioactive	262 (25) ab	97 (11) ab
B3	270 (23) ab	98 (10) ab
B5	272 (59) ab	100 (26) ab
B7	235 (36) b	88 (17) b
B10	226 (42) b	83 (17) b
B15	220 (17) b	84 (8) b

**Table 2:** Degree of conversion (%) for control and bioactive dental adhesives at 6 and 12 months (mean and standard deviation). The letters indicate statistical comparisons among mean. In the same column, values followed by the same lowercase letter do not present statistical differences (*p* > 0.05). In the same row, values followed by the same uppercase letter do not present statistical differences (*p* > 0.05).

Group	Degree of Conversion %		
	24 h	6 Months	12 Months
C—Control	87 (3) abA	89 (3) aA	88 (2) aA
B—Bioactive	70 (7) bA	89 (7) aA	87 (5) aA
B3	69 (2) bB	93 (1) aA	92 (3) aA
B5	85 (10) abA	91 (10) aA	92 (7) aA
B7	93 (10) aA	96 (6) aA	94 (7) aA
B10	91 (14) aA	99 (12) aA	91 (4) aA
B15	70 (4) bA	88 (7) aA	88 (6) aA

**Table 3:** Bond Strength (MPa) for control and bioactive dental adhesives at 6 and 12 months (mean and standard deviation). The letters indicate statistical comparisons among mean. In the same column, values followed by the same lowercase letter do not present statistical differences (*p* > 0.05). In the same row, values followed by the same uppercase letter do not present statistical differences (*p* > 0.05).

Group	Bond Strength (MPa)		
	24 h	6 Months	12 Months
C—Control	12 (4) aA	9 (2) aA	6 (4) bB
B—Bioactive	10 (3) aA	9 (4) aA	8 (3) aA
B3	9 (3) aA	10 (3) aA	9 (4) aA
B5	11 (2) aA	10 (2) aA	9 (4) aA
B7	13 (3) aA	9 (3) aA	8 (3) aA
B10	12 (4) aA	10 (3) aA	9 (5) aA
B15	9 (3) aA	10 (4) aA	9 (3) aA

## Data Availability

The original contributions presented in this study are included in the article. Further inquiries can be directed to the corresponding author.

## Supplementary Materials

The following supporting information can be downloaded at: https://www.mdpi.com/article/10.3390/ma18194433/s1, Figure S1: Mean and standard deviation of sorption (μg/mm^3^) of the experimental and control materials; Figure S2: Mean and standard deviation of solubility (μg/mm^3^) of the experimental and control materials; Figure S3: Mean and standard deviation of degree of conversion (%) of the experimental and control materials measured at 24 h, 6 and 12 months; Figure S4: Mean and standard deviation of degree of conversion (%) of the experimental and control materials measured at 24 h, 6 and 12 months. (*) indicates the only material that is statically different from the others after 12 months of storage.
