# Supplementary material for: Improving Bonding Durability in Dental Restorations: The Impact of Bioactive and Reinforcement Particles on Universal Adhesives
Source: Materials (Basel). 2025 Sep 23;18(19):4433. doi: 10.3390/ma18194433 (PMC12525280; doi:10.3390/ma18194433)
Supplement: Supplementary file 1 [file materials-18-04433-s001.zip › materials-3804200-supplementary.pdf]

Supplemental Materials

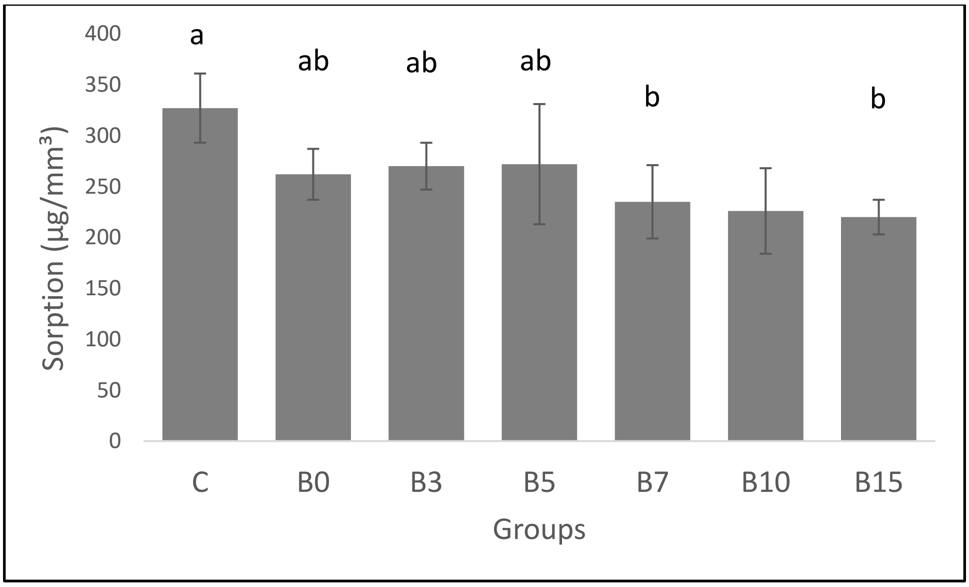

Figure S1: Mean and standard deviation of sorption (µg/mm³) of the experimental and control materials.

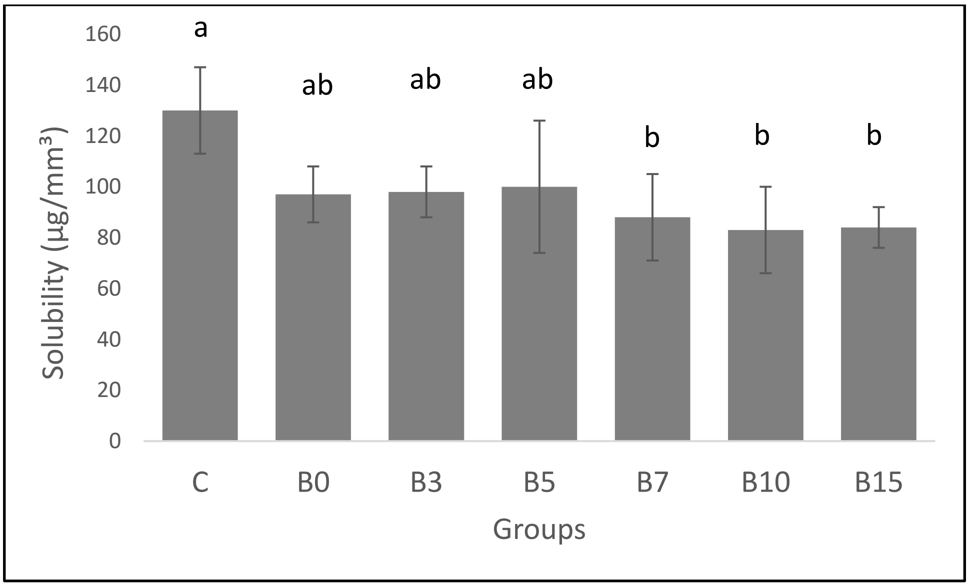

Figure S2: Mean and standard deviation of solubility (µg/mm³) of the experimental and control materials.

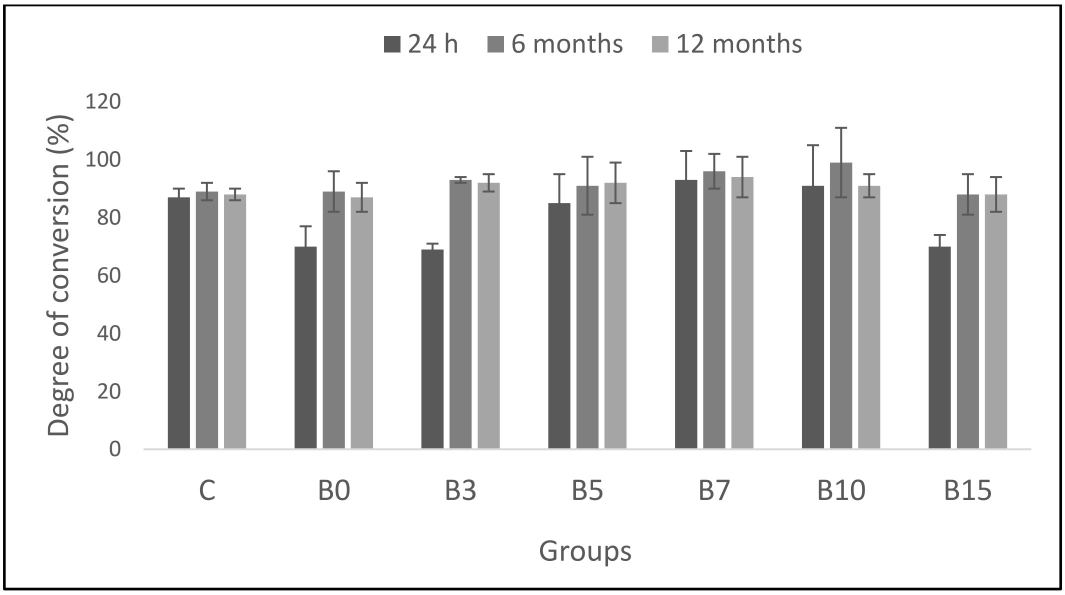

Figure S3: Mean and standard deviation of degree of conversion (%) of the experimental and control materials measured at 24h, 6 and 12 months.

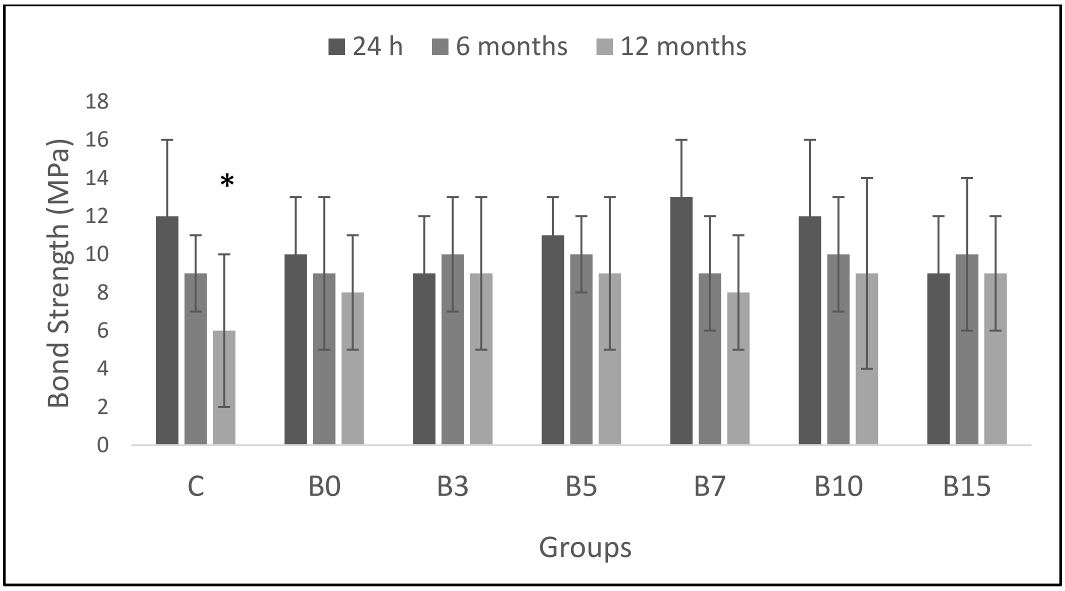

Figure S4: Mean and standard deviation of degree of conversion (%) of the experimental and control materials measured at 24h, 6 and 12 months. (*) indicates the only material that is statically different from the others after 12 months of storage.
